# Self organising hypothesis networks: a new approach for representing and structuring SAR knowledge

**DOI:** 10.1186/1758-2946-6-21

**Published:** 2014-05-08

**Authors:** Thierry Hanser, Chris Barber, Edward Rosser, Jonathan D Vessey, Samuel J Webb, Stéphane Werner

**Affiliations:** 1 Lhasa Limited, Leeds, UK

**Keywords:** Machine learning, Knowledge discovery, Data mining, SAR, QSAR, SOHN, Interpretable model, Confidence metric, Hypothesis Network

## Abstract

**Background:**

Combining different sources of knowledge to build improved structure activity relationship models is not easy owing to the variety of knowledge formats and the absence of a common framework to interoperate between learning techniques. Most of the current approaches address this problem by using consensus models that operate at the prediction level. We explore the possibility to directly combine these sources at the knowledge level, with the aim to harvest potentially increased synergy at an earlier stage. Our goal is to design a general methodology to facilitate knowledge discovery and produce accurate and interpretable models.

**Results:**

To combine models at the knowledge level, we propose to decouple the learning phase from the knowledge application phase using a pivot representation (lingua franca) based on the concept of hypothesis. A hypothesis is a simple and interpretable knowledge unit. Regardless of its origin, knowledge is broken down into a collection of hypotheses. These hypotheses are subsequently organised into hierarchical network. This unification permits to combine different sources of knowledge into a common formalised framework. The approach allows us to create a synergistic system between different forms of knowledge and new algorithms can be applied to leverage this unified model. This first article focuses on the general principle of the Self Organising Hypothesis Network (SOHN) approach in the context of binary classification problems along with an illustrative application to the prediction of mutagenicity.

**Conclusion:**

It is possible to represent knowledge in the unified form of a hypothesis network allowing interpretable predictions with performances comparable to mainstream machine learning techniques. This new approach offers the potential to combine knowledge from different sources into a common framework in which high level reasoning and meta-learning can be applied; these latter perspectives will be explored in future work.

## Background

Developing robust Structure Activity Relationship (SAR) models for a given endpoint requires a good understanding of the interaction between the chemical compound and the targeted biological system. This knowledge can be extracted from experimental observations compiled in the form of datasets. Thorough analysis of these datasets, either manually by expert scientists (Expert Learning) or automatically by computer algorithms (Machine Learning) [1], can provide a better understanding of the structure activity relationships. The resulting knowledge can be captured in the form of SAR rules, and implemented in expert systems, or lead to quantitative statistical models (QSAR). In both cases the underlying knowledge becomes a central asset for further exploring the endpoint’s mechanism or predicting the behaviour of unseen compounds.

Depending on the learning method used, the resulting knowledge will present different qualities associated with various use cases. Although the accuracy of SAR and QSAR models has for a long time been the primary concern and used as the main performance measure [2], there are other important facets amongst which are:

The transparency of the model and the interpretability of the predictions they provide [3-5]

The ability to estimate the confidence in an individual prediction [6,7]

The possibility to define an applicability domain of the model [8-12]

The proportion of structures for which an actual classification can be made (coverage)

The ability to provide supporting evidence (e.g. real world examples)

**Figure 1:**
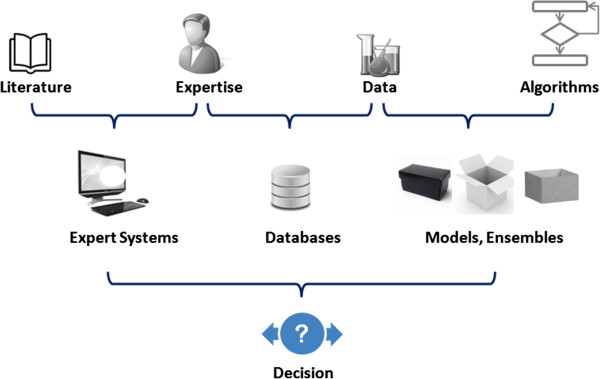
**Decision supporting tools.** End users often rely on various combinations of tools and knowledge representations to build a decision.

All these properties have gained importance recently especially in the context of risk assessment where they are partially covered by the Organisation for Economic Cooperation and Development (OECD) guidelines [13].Addressing all of these aspects in parallel is particularly challenging and as a result it is difficult to design the perfect model that resolves all of them simultaneously. Fortunately the end users can combine their own expertise with different tools and sources of knowledge (expert systems, statistical models, databases, etc.) in order to build a well informed decision (Figure 1).

Whilst there is a large choice of such tools, it is difficult to build a framework to combine their knowledge and help leverage the potential synergy between different models. For instance interoperability between different approaches is challenging due to a lack of unified knowledge representation and there is no standard way to provide overall decision support. The main reason for this is that we are lacking a common language that would help us take advantage of different forms of knowledge within a common framework.

**Figure 2:**
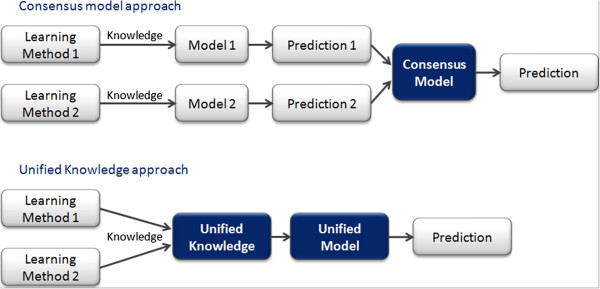
**Consensus model vs. Unified knowledge.** Two ways of combining different learning methods; the consensus approach by merging the prediction (late stage) and the unified knowledge approach by combining the knowledge (early stage).

A common practice is to combine the predictions of several models into an overall outcome using consensus based reasoning. This approach has sometimes proven to provide better performance than the separate individual models [14]. In this consensus method, models are combined at a late stage i.e. the prediction phase. It is legitimate to investigate if there is room for further improvement by merging these models at an earlier stage, i.e. directly at the knowledge level and in so doing, leverage any form of synergy prior to predicting (Figure 2). Furthermore, if we manage to combine sources of knowledge rather than final predictions we can develop a common library of algorithms that can be applied to this higher level abstraction. With this question in mind we initiated a project that aims to build a common knowledge platform along with algorithms to facilitate knowledge discovery and produce interpretable and accurate predictions. We present here our first step towards this goal in the form of a novel way to abstract and organize knowledge which will become the cornerstone of this new approach.

## Method

**Figure 3:**
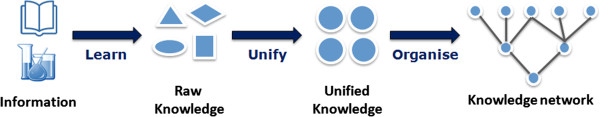
**The SOHN methodology.** Different sources of knowledge are unified using a common representation based on the concept of hypothesis. The hypotheses can be organised into a hierarchical network to capture the knowledge in a standardised way.

The proposed approach is based on 3 key steps. First, derive knowledge from different sources of information (experimental data, expert learning, machine learning). Secondly, unify the different knowledge representations. Finally, organise the unified knowledge in a way that captures its generalisation hierarchy and facilitates the design of efficient prediction algorithms (Figure 3).

Different learning methods will usually use different knowledge formats which make the knowledge merging process difficult. One possible solution is to define a common representation (‘lingua franca’) in order to combine the knowledge produced by various learning techniques. We also want the final global model to be transparent and thus the common representation must be composed of interpretable elements of knowledge. For that purpose we introduce the concept of hypothesis: a simple and interpretable knowledge unit. In the first step we collect knowledge relevant to the target endpoint using several sources and we break down this knowledge into a collection of hypotheses. Once the hypotheses have been generated we can organise them into a hierarchical structure that captures the different level of generalisation of the knowledge. This structure is automatically updated each time a new hypothesis is inserted following a specific algorithm that ensures the overall consistency of the knowledge. The structure is called a Self-Organising Hypothesis Network or SOHN.

Finally, powerful algorithms can take advantage of the SOHN for analysing the underlying knowledge and perform accurate and transparent predictions. These algorithms no longer depend on the initial specific learning method or knowledge representation; hence they can be mutualised across the different mining techniques. In this higher level of processing, the unified and structured knowledge representation can be fully leveraged.

### Knowledge unification

**Figure 4:**
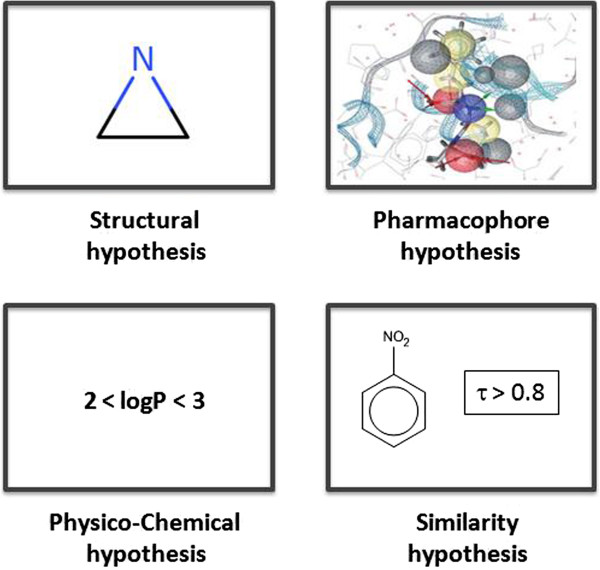
**Different types of hypotheses.** Hypotheses can capture different attributes of a compound relevant to the target endpoint. The SOHN abstraction can handle different types of hypotheses within the same SOHN.

The central concept of our approach is the hypothesis. A hypothesis defines the scope of a class of compounds sharing a SAR trend for a given endpoint. From a classification viewpoint, a hypothesis defines a class of compounds in the chemical space that fully or partially separates the endpoint classes. A hypothesis can be seen as a local model representing a knowledge unit.In practice, hypotheses can take different forms depending on the molecular information that they take into account (Figure 4). For instance structural hypotheses can represent a class of compounds containing a given functional group or more generally a structural pattern. Other hypotheses may be based on physico-chemical or pharmacophoric properties of the structure. There is no restriction on how the scope of hypotheses may be defined, for instance, we can even define a hypothesis that matches all the structures that are similar to nitro-benzene with a Tanimoto index greater than 0.8. Hypotheses are simply an abstract way to express SAR assumptions.

Although there are no predefined rules regarding their scope, all hypothesis types must provide a minimum of functionalities, i.e. fulfil a contract, in order to build our unified knowledge framework. The contract for a given type of hypothesis contains only three clauses.

#### *Instance matching*

We must be able to check if a hypothesis covers (i.e. subsumes) a given structure. We say that a hypothesis *h* covers a structure *s* if *s* belongs to set of structures that *h* matches. The latter is also known as the scope of *h* and this relationship is noted: ***s *** **∈ *Scope*** **(*h*)**.

For instance in the case of a structural hypothesis this clause can be implemented using the presence or absence of a structural fragment.

#### *Domain matching*

We need to know if a given compound belongs to the applicability domain of a given type of hypothesis. The applicability domain of a hypothesis *h* is the set of structures for which the hypothesis can be reliably evaluated. This relationship is noted: ***s *** **∈ *Domain*** **(*h*)**.

For instance for structural hypotheses, a reference dictionary of common moieties in the target chemical space can be built and this clause could state that a structure belongs to the applicability domain if all its atoms and bonds are covered by at least one fragment from this dictionary.

#### *Similarity measure*

Each type of hypotheses must provide a way to compute a symmetrical and normalised index of similarity (ranging from 0 to 1) between two instances of the input space. The similarity index between two structures *s*_1_ and *s*_2_ given a hypothesis *h* is noted: ***Similarity*** **(*h*, *s***_**1**_**, *s***_**2**_**)**.

In the case of structural hypotheses it is, for instance, possible to use a fingerprint based Tanimoto index to implement this clause.

These simple functionalities are all that is required in order to define a new type of hypothesis.

Once a hypothesis type has been designed and its contract implemented we can derive two fundamental properties of a given hypothesis.

#### *Coverage*

Given a reference dataset ***D*** (set of instances) the coverage ***C*** of a hypothesis ***h*** is the subset of instances for which the hypothesis applies (intersection between the scope of *h* and the dataset).

$$C\left(h,D\right)=\left\{s|s\in D,s\in \mathrm{Scope}\left(h\right)\right\}$$

#### *Signal*

Given a reference dataset ***D*** (collection of instances) the signal of a hypothesis ***h*** for a given class (or label) ***y*** is defined as follows:

$$\begin{array}{c} \begin{array}{c} S_{y}\left(h,D\right)=\frac{L\times n_{y}\left(h,D\right)-N\left(h,D\right)}{\left(L-1\right)\times N\left(h,D\right)}\\ n_{y}\left(h,D\right)=\left|\left\{s\;\in \;C\left(h,D\right);s\;\in \;y\right\}\right|=\mathrm{number}\;\mathrm{of}\;\mathrm{instances}\;\mathrm{labelled}\;y\\ N\left(h,D\right)=\underset{y\in Y}{\sum }n_{y}\left(h,D\right)=\left|C\left(h,D\right)\right|=\mathrm{number}\;\mathrm{of}\;\mathrm{instances}\;\mathrm{covered}\;\mathrm{by}\;h\\ L=\left|Y\right| \end{array} \end{array}$$

where ***Y*** is the set of classes or labels for the target endpoint.

The signal indicates the deviation in the distribution of the classes (compared to a balanced distribution n) amongst the covered examples. A signal value of 0 for a class *y* indicates that the hypothesis does not discriminate this class from other classes. A signal value of 1 means that the hypothesis covers only examples from the class *y* and is therefore fully discriminating.

From a learning viewpoint, a good hypothesis is a one that combines a strong signal and a wide coverage. Such a hypothesis represents an interesting concept related to the target endpoint.

**Figure 5:**
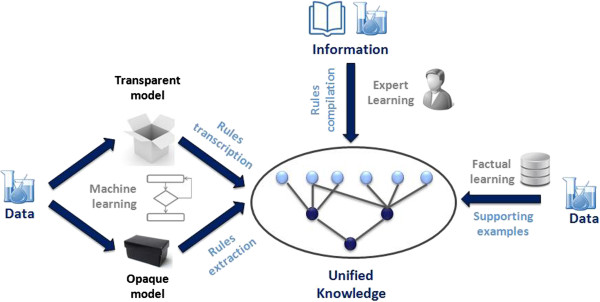
**Combining different source of knowledge.** Different sources of knowledge can be merged into a structured and unified model. This permits to combine different learning techniques (expert learning, machine learning and facts).

We can now consider different sources providing elements of knowledge in the form of simple hypotheses (Figure 5). For instance, human experts can contribute knowledge in the form of structural alerts. Automated machine learning can also be applied in the knowledge extraction phase with transparent models providing interpretable rules that can be directly transcribed into a set of corresponding hypotheses. This applies to machine learning techniques like Inductive Logic Programming [15] and Decision Trees [16] (DT). In the case of less transparent models, like Support Vector Machines [17] (SVM) or Random Forests [18] (RF) etc., it is possible in some cases to apply automatic rule extraction techniques and transform these identified rules into hypotheses. Finally experimental data represent factual knowledge and each observation becomes a very specific hypothesis (only one instance covered and a signal value equal to 1). Example based hypotheses usually provide a very poor level of generalisation but they represent experimental evidence which makes them extremely valuable.

### Knowledge organisation

**Figure 6:**
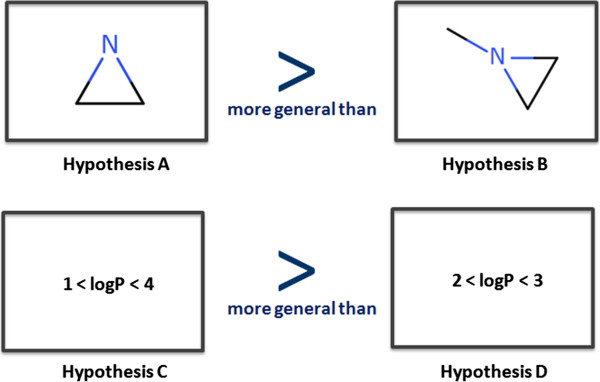
**Ordering hypotheses according to their degree of generalisation.** Hypotheses can be ordered according to their level of generalisation. Here, hypotheses A is more general than hypothesis B (A is a substructure of B). Similarly C is more general than D.

**Figure 7:**
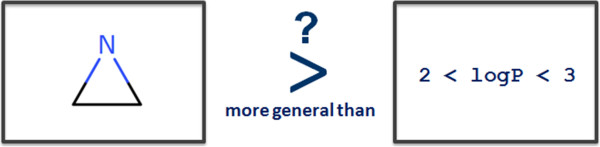
**Difficulty in ordering hypotheses of different types.** Comparing the level of generalisation between hypotheses of different types is not trivial and in some cases impossible when the underlying molecule attributes are not directly comparable.

Once hypotheses have been gathered from one or several sources they can be organised into a useful structured knowledge representation. Hypotheses convey very local models for a given endpoint, these units of knowledge can be organised into a meaningful hierarchy. From the knowledge mining perspective the most important relationship between hypotheses is their degree of generalisation. For instance, in Figure 6, hypothesis A is more general than hypothesis B since all the structures covered by B are necessarily covered by A. Similarly the logP based hypothesis C is more general then D.In both cases it is easy to understand how to define the order since we compare hypotheses of the same types. We intuitively perform a substructure comparison between A and B and find A included in B and thus can state that it is more general. We can also perform a logP range comparison between C and D and infer that C is more general than D because its logP scope includes the range of values allowed in D. However when we try to compare hypotheses of different types, the ranking becomes more difficult and less intuitive. For instance in Figure 7, which hypothesis is the more general?

**Figure 8:**
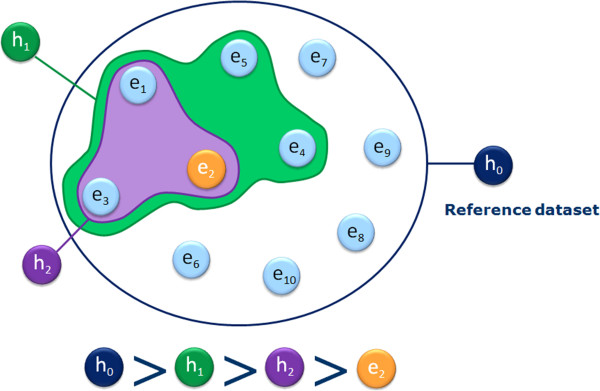
**A more universal way to order hypotheses.** Using a reference dataset we can define the generalisation hierarchy based on the coverage of the hypotheses. Thus we do not depend on the type of information handled and we can compare hypotheses of different types.

It is not possible to answer the question by simply comparing the two hypotheses. Instead we need a way to define generalisation independently from the hypothesis type. In our approach we propose to use a reference dataset so we can define a generalisation order based on the coverage of the hypotheses within this dataset. The method is illustrated in Figure 8 with a reference dataset containing 10 examples (e_1_ to e_10_). Let us assume that a hypothesis h_1_ covers examples e_1_ to e_5_ and a second hypothesis h_2_ applies to the examples e_1_, e_2_ and e_3_. We can now intuitively infer that h_1_ is more general than h_2_ since all the examples covered by h_2_ are also covered by h_1_. Here we simply use the first clause (matching) of the hypothesis contract given a reference dataset *D*.

$$\begin{array}{c} h_{1}>h_{2}\;\mathrm{iff}\;\forall e\in \mathrm{Scope}\left(h_{2}\right)\Rightarrow e\;\in \;\mathrm{Scope}(h_{1}).\;\Leftrightarrow \;C(h_{2},D)\subset C(h_{1},D)\\ \;\left(\mathrm{where}>\mathrm{means}‘\mathrm{more}\;\mathrm{general}\;\mathrm{than}’\right) \end{array}$$

The definition implies that if two hypotheses have identical coverage, they do not have a parent/child relationship.

**Figure 9:**
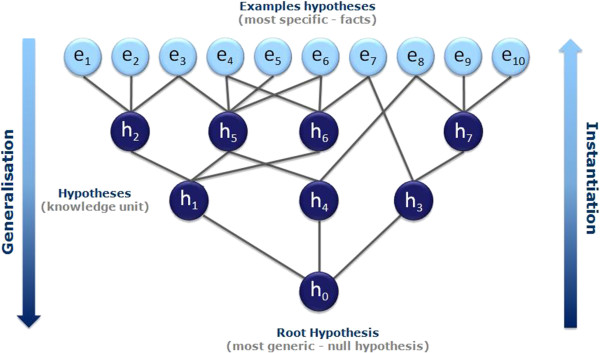
**Self Organising Hypothesis Network.** Using the ‘is more general than’ relationship a collection of hypotheses can be organised into a hierarchical network from the most generic (the root) to the most specific (the examples).

Note that there are two extreme cases of hypotheses. First the whole chemical space can be seen as the resulting coverage of a hypothesis that matches any structure and therefore represents the most general hypothesis possible; we call it the root hypothesis h_0_. On the other end of the spectrum, each example is a form of an ultimately specific hypothesis that covers only itself. Thus in terms of generalisation, in our illustration h_0_ > h_1_ > h_2_ > e_2_. Pairs of hypotheses that do not have subset/superset relationships are said to be ‘*incomparable’* (e.g. h_1_ and h_4_ in Figure 9).Having defined a universal way to compare the hypotheses according to their degree of generalisation, we can now organise them into a hierarchical network from the most general (the root) to the most specific (the reference dataset examples) creating a top-down generalisation axis and a bottom-up instantiation axis (Figure 9).

We have developed an algorithm that automatically updates the network when a new hypothesis is inserted (or removed) and we call the resulting data structure a Self-Organising Hypothesis Network (SOHN).

An implementation of the algorithm will typically also allow making decision about inserting or not a hypothesis based on the information gain depending on the insertion point.

Although the structure of a SOHN is very close to a Galois lattice used in Formal Concept Analysis [19] (FCA) and hypotheses play here a similar role to concepts in FCA, there are fundamental differences between the two approaches. Whereas in FCA, concepts are derived from the examples, in the SOHN approach hypotheses are not necessarily inferred from the reference dataset. For instance a hypothesis can be designed by a human expert. In the SOHN method, examples in the reference dataset are principally used to organise and support the hypotheses. Furthermore, in FCA the relation ‘is more general than’ is defined at the attribute level (and by extension, at the implied covered example level) and assumes that all the concepts work in the same predefined attribute space. In the SOHN approach the relation depends only on the example coverage and, it is therefore a less constrained methodology. This allows us to combine different sources and types of hypotheses and subsequently project them on an independent reference dataset. In FCA the example set and the inferred concepts are interdependent by principle whereas in the SOHN approach example and hypotheses are inherently independent although they are often related in practice as a consequence of the knowledge extraction process. Nevertheless the SOHN method can also benefit from some of the FCA techniques when these differences are not critical, the exploration of such opportunities is not in the scope of this article.

It is important to note that the reference dataset is required to establish the hypothesis hierarchy and that it becomes part of the final network. Different reference datasets may induce different hierarchies of hypotheses; this reflects the fact that the level of generalisation is not intrinsic to a hypothesis but depends on the chemical space in which the hypotheses are applied. Also, the reference dataset is not to be mistaken with a training dataset.

**Figure 10:**
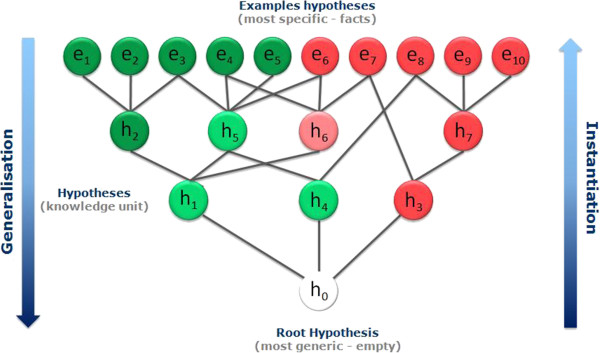
**SOHN with activity information.** When overlaying the signal of the hypotheses for a given endpoint, the SOHN becomes an interesting SAR analysis tool.

Once the SOHN has been constructed for a collection of hypotheses and a given reference dataset, it is possible to analyse the signal and coverage of each hypothesis. In Figure 10 each example is colour coded; green for positive examples e_1_-e_5_ and red for negative examples e_6_-e_10_. The colours for the hypotheses correspond to the intensity of their signal. From a learning perspective we expect the hypotheses to have a good combination of strong signal and wide coverage. These two parameters are usually antagonistic and we expect the different sources of knowledge to optimise knowledge extraction and provide “good” hypotheses during the learning phase. The quality of the knowledge contained in a SOHN will therefore depend on the learning performance of the individual hypotheses providers (not the SOHN methodology itself); additional knowledge may emerge from the combination and organisation of this knowledge within the SOHN as the result of a synergetic effect.

The resulting SOHN and its associated information network is a computer friendly data structure and this knowledge structure can be efficiently used in the context of SAR analysis and for the prediction of unseen compounds.

## Results

### An example of SOHN for the mutagenicity endpoint

**Figure 11:**
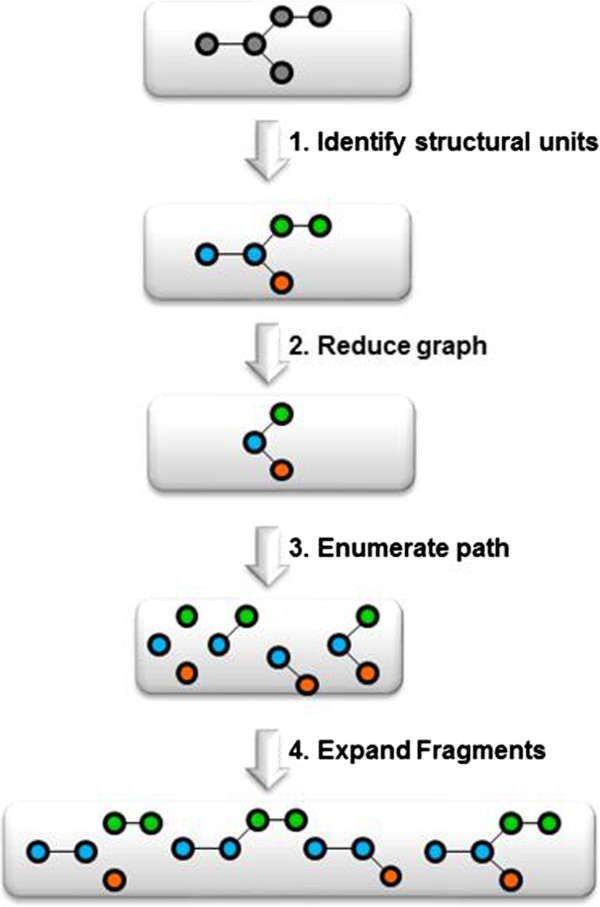
Fragmentation methodology.

**Figure 12:**
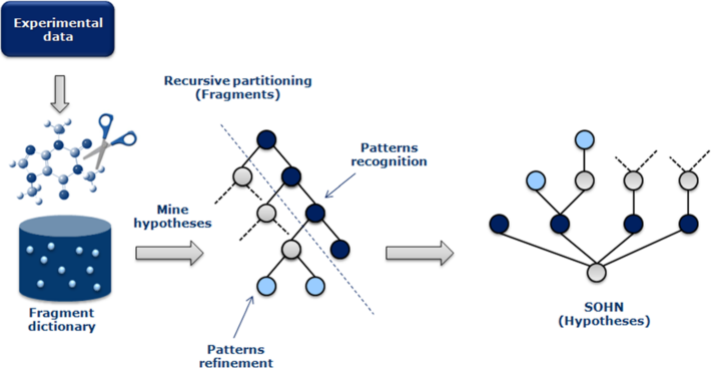
**Example of workflow used to convert a dataset into a SOHN representation.** Structures in the dataset are first fragmented. The knowledge is then mined using a recursive partitioning approach to identify information-rich fragments which are converted into corresponding structural hypotheses. The input dataset is also used as the reference example dataset to build the SOHN.

**Figure 13:**
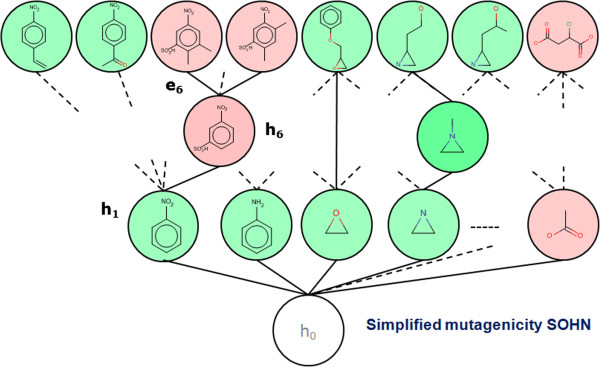
**Example of SOHN for the mutagenicity endpoint.** A simplified view of the SOHN network build from 8,201 structures for the mutagenicity endpoint. In this representation, fragment hypotheses are open structures whereas example hypotheses are closed structures.

This section illustrates a simple example of a SOHN network for hypotheses designed to capture knowledge about the toxicological mutagenicity endpoint. The knowledge was mined from a dataset of 8,201 structures distributed over 2 classes, 50% mutagen, and 50% non mutagens. Only 2 sources of hypotheses were used. The first source is the dataset itself, providing the top level example hypotheses and the second is a machine learning algorithm, namely a Decision Tree, producing structural hypotheses. Decision Trees were chosen because they provide directly interpretable nodes which are easy to convert into hypotheses. For this purpose the 8,201 structures have been fragmented into ~20,000 fragments using an in-house algorithm based on a reduced feature graph [20,21]. Rather than to fragment the original molecule, our fragmentation method first builds an intermediate reduced graph where all the nodes represent a structural unit of the original molecule. The scope of a structural unit is flexible and can be adjust to different use-cases (chemical reactivity, pharmacophoric abstraction, etc.). Structural units can for instance represent single atoms and bonds, functional groups, rings, fused rings, etc. Once the reduce graph has been constructed we fragment this graph using a combination of circular and linear path enumeration. Each fragment generated from the reduced graph is expanded back to a molecular fragment graph. The depth of the path enumeration can be configured. This fragmentation method allows us to take advantage of an exhaustive path enumeration without the risk of breaking logical units within the molecules (Figure 11).Finally, fragments present in at least 4 examples, were used as 2D structural descriptors to build a Decision Tree resulting in 400 nodes. Fragments retained as nodes in the Decision Tree represent good hypothesis candidates and all 400 nodes were converted into the corresponding structural hypotheses. Note that the Decision Tree algorithm used ensures that each decision node corresponds to a more specific hypothesis than its parent allowing the Decision Tree hierarchy to be rediscovered during the SOHN building process. In order to optimise the knowledge extraction process hypotheses can be further filtered against different criteria like information gain, minimum coverage, etc. Note that we are not interested in the Decision Tree as a final model, we used this machine learning technique only to identify relevant structural patterns that we eventually capture in the form of SOHN hypotheses (Figure 12).Finally the 400 hypotheses have been organised into a SOHN following the methodology described earlier. At this point we are no longer dependent upon the source of the hypotheses i.e. we become agnostic of the learning method used. A simplified version of the resulting SOHN is shown in Figure 13.

### SOHN as SAR analysis tool

In the SOHN built in the previous section, the general hypotheses (first level above the root) with a positive signal (mutagenic activity) correspond to common structural alerts for this endpoint. Indeed, moieties like aromatic nitro groups, aromatic amines, epoxides, and aziridines are known toxicophores. The presence of these expected patterns means that the Decision Tree algorithm, using the fragment descriptors, succeeded in mining relevant knowledge from the dataset. These hypotheses represent general rules covering a relatively large proportion of the dataset and are therefore key elements of knowledge for the studied endpoint. At a more specific level, hypothesis h_6_ tells us that the mutagenic effect of the aromatic nitro group (h_1_) can be mitigated by the presence of a sulfonic acid group in the meta position. h_6_ is a child of h_1_ and therefore represents a refinement rule (more specific hypothesis). The signal contrast between h_1_ and h_6_ clearly indicates an interesting activity change and the structural transition from h_1_ to h_6_ provides a useful explanation.

More generally each path in the SOHN contains rich SAR information helpful in many contexts such as:

• activity cliff detection [22]

• matched molecular pairs identification [23]

• knowledge discovery and refinement

• lead optimisation

The most specific hypothesis (the observed data points) provide underlying supporting evidence. For instance hypothesis h_6_ is supported by several examples in the reference dataset among which e_6_.

**Figure 14:**
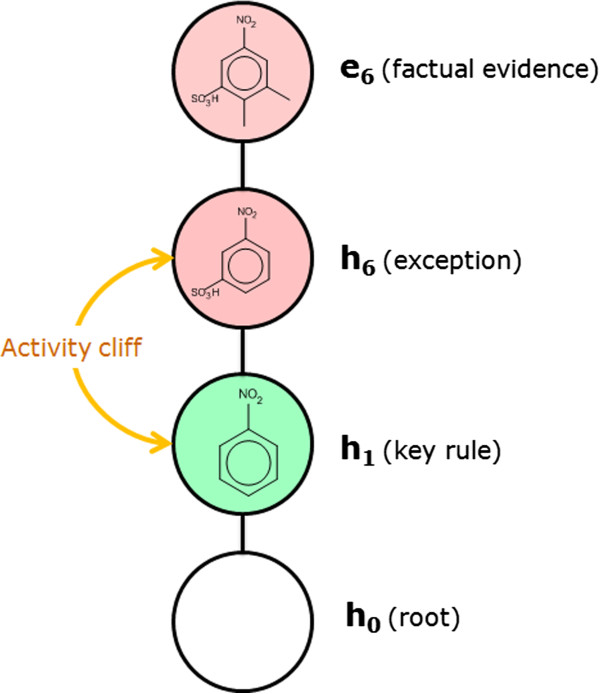
**Information within the SOHN paths.** Each path in the SOHN provides structured knowledge about the SAR that can be used to discover activity cliffs, suggest lead optimisation or interpret predictions.

The SOHN structure provides a rich representation of the knowledge associated with a given endpoint and facilitates the SAR analysis. When graphically displayed, SOHN provide useful visual information about the SAR landscape of the reference dataset similarly to SAR trees or pathways [24] and Scaffold trees [25] visualisations. In the latter, the information is structure-centric whereas in SOHN it is hypothesis centric. Hypotheses are organised in SAR paths going from generic key rules to factual evidence *via* intermediate hypotheses that capture important refinement factors. Each path from the root hypothesis to more specific hypotheses and eventually to real examples describes a SAR route that can contribute to an explanation of the activity of these examples (Figure 14).

It is noteworthy that even though, in this case, the hypotheses were generated using a Decision Tree, their organisation inside the SOHN has a network structure and not a tree based structure. Indeed specific hypotheses may have several more generic parents and *vice versa* (as implied in Figures 9 and 13).

### SOHN as a predictive model

When human experts try to predict the activity of an unseen compound (query compound) they usually analyse the chemical structure and search for parts of their knowledge that can be applied to the structure. The expert builds up a collection of hypotheses about the activity of the query based on these selected elements of knowledge. When some elements are contradictory, the expert will weigh the retained hypotheses according to their relevance to the analysed compound.

The SOHN approach uses exactly the same method. The strongly hierarchical structure of the SOHN allows us to define a deterministic and optimised algorithm to explore all the possible hypotheses that can apply to a new unseen compound and to select the most relevant ones. This first step can be achieved using the following algorithm:

The most relevant hypotheses for a query compound *x* can be found by calling the function for the root of the network:

$$\mathrm{relevantHypothese}s_{x}=\mathrm{FindRelevantHypotheses}\left(h_{0},x\right).$$

**Figure 15:**
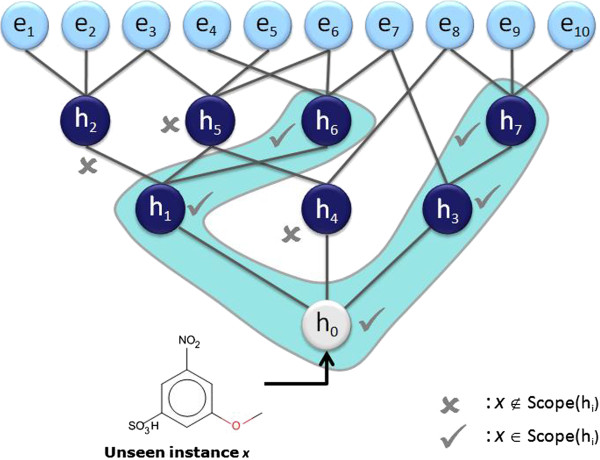
**Example of prediction analysis.** To find the most relevant knowledge that will contribute to a prediction, the SOHN algorithm recursively searches the network for the most specific hypotheses that apply to the query compound, starting from the root. In the current case h_6_ and h_7_ have been identified as the most specific hypotheses (none of the examples matches the query).

**Figure 16:**
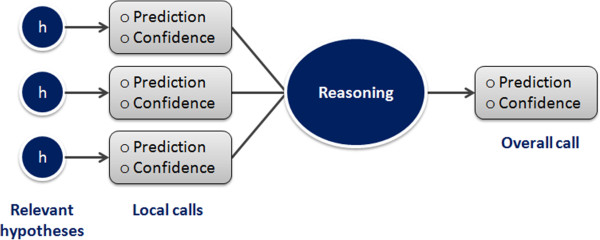
**Combining local hypotheses prediction into a global prediction.** Local prediction based on individual hypotheses are combined into an overall call based on a reasoning method that can be adapted to the use case (screening, lead optimisation, risk assessment, etc.).

The algorithm recursively explores the SOHN network starting from its root and identifies the most specific hypotheses that apply to the query compound (first clause of the hypothesis contract) and for which the compound is in the applicability domain (second clause of the contract). The resulting hypotheses represent the most relevant knowledge for predicting the activity of the compound. Each hypothesis retained corresponds to the most local model that implements a partial contribution of the knowledge. Figure 15 describes a virtual example of SOHN exploration leading to two final hypotheses h_6_ and h_7_. The ability for the model to consider several hypotheses simultaneously is a very interesting feature (often absent in other methods). Indeed molecules may contain several causes of activity or inactivity and it is important for a good prediction to take into account all these factors. The identification of all the relevant effects is also valuable information in a decision process and will help the expert assess the prediction based on these factors.The selected hypotheses and the corresponding paths from the root provide the necessary knowledge to build an informed decision about the class of the query. This knowledge is composed of hypotheses and factual evidence which can be used in different ways to construct a final overall prediction. To this end, we have developed a prediction methodology that takes advantage of all the available information in the identified hypotheses to build an accurate prediction along with a confidence level and supporting examples (Figure 16). This method is divided into two main steps:

1. Construct individual hypothesis predictions which will contribute to the overall call. In this step each hypothesis will predict a class for the query compound. Each hypothesis has a confidence level and a set of supporting examples attached to its ‘local’ prediction.

2. Combine the hypotheses prediction into a final overall call by taking into account the individual hypothesis predictions and their respective confidence levels. Different ways to combine these hypotheses can be considered depending on the desired use case.

### Individual hypothesis prediction (local prediction)

**Figure 17:**
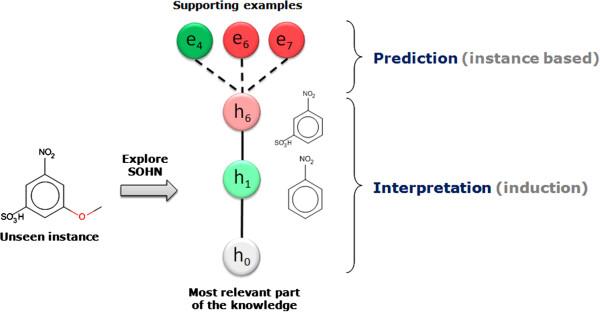
**Prediction and interpretation analysis.** Each hypothesis contributing to the prediction provides information to support the actual prediction (the supporting examples) and interpret the outcome of this prediction (the path from the root to the hypothesis); having simple and interpretable hypotheses in the first place will contribute to the transparency of the model.

The path from the root to the hypothesis is used as supporting knowledge to explain the prediction’s outcome whereas the actual prediction is built from the reference dataset examples covered by the hypothesis using a kNN [26] algorithm (Figure 17).

**Figure 18:**
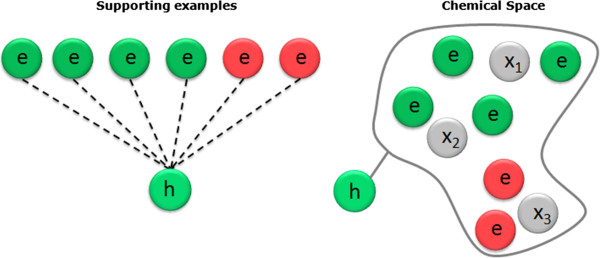
**Accounting for the SAR landscape in the prediction confidence.** The local prediction for an individual hypothesis uses a kNN model based on the supporting examples. This allows accounting for the SAR landscape in the corresponding region of the chemical space. In this illustration, the query *x*_1_ will be predicted positive with high confidence, *x*_2_ positive with less confidence and *x*_3_ will be predicted negative overruling the hypothesis which can be ignored.

This instance-based prediction takes into account the local SAR ‘landscape’ (distribution of classes within the supporting examples) and identify situations where the query compound might occupy a region of the chemical space which is not in agreement with the hypothesis. In Figure 18, the query compound *x*_1_ will be predicted positive with high confidence, and *x*_2_ will be positive with less confidence than for *x*_1_. The query *x*_3_ will be predicted negative in contradiction with the hypothesis; in this case the hypothesis is said to be overruled and can be ignored.

To make a local prediction from a hypothesis, first the k (k = 10) nearest neighbours to the query are identified amongst the supporting examples of the hypothesis. Then a weighted prediction signal is computed using the similarity of the examples with the query compound as the weighting factor. The square root of the similarity has been identified as a good weighting using cross validation and the following measure is used:

(1) $$w_{i,x}=\sqrt{\mathrm{Similarity}\;\left(x,e_{i}\right)}$$

(2) $$ws_{h,x}=\frac{\sum_{i=1}^{k}w_{i,s}\times s_{i}}{\sum_{i=1}^{k}w_{i,x}}$$

Where:

*w*_*i,x*_ is the weight assigned to the nearest neighbour *e*_i_ given the query compound *x*.

*Similarity*(*x,e*_*i*_*)* is the similarity index between the query *x* and the nearest neighbour *e*_*i*_ (using the third clause of the hypothesis contract).

*s*_*i*_ is the instance signal (its class) of the nearest neighbour *e*_*i* ;_*s*_*i*_ = -1 if e_i_ is a negative example; *s*_*i*_ = +1 if *e*_*i*_ is a positive example. For the sake of simplicity, the scope of this article will only address binary classification. However the methodology supports continuous values for the signal and more generally, multiple classes using signals distributed over more than 2 classes. Regression problems can also be considered using a similar formalism.

Secondly we further moderate the weighted signal to account for the average distance of the nearest neighbours to the query compound. The final prediction signal for the hypothesis h can be written:

(3) $$S_{h,x}=ws_{h,x}\times \frac{\sum_{i=1}^{k}w_{i,x}}{k}$$

Thus replacing *ws*_*h*,*x*_ from Equation 2

(4) $$S_{h,x}=\frac{\sum_{i=1}^{k}w_{i,x}\times s_{i}}{k}$$

The sign of the final signal *S*_*h,x*_ is used to classify the compound *x* according to the hypothesis *h* and the absolute value of the signal is used to measure the confidence in the prediction. The closer to +1 or -1 the more confident we are (according to the current hypothesis) that the compound is positive or negative respectively and the closer to 0 the lower the level of confidence attached to the local prediction. A signal of 0 is seen as equivocal.

$$\begin{array}{c} \mathrm{clas}s_{h,x}=\left\{\begin{array}{c} \mathrm{positive}\;\mathrm{if}\;s_{h,x}>0\\ \mathrm{negative}\;\mathrm{if}\;s_{h,x}<0\\ \mathrm{equivocal}\;\mathrm{if}\;s_{h,x}=0 \end{array}\right)\\ \mathrm{confidenc}e_{h,x}=\left|s_{h,x}\right| \end{array}$$

The confidence ranges from 0 (equivocal) to 1 very confident (or certain in the case of an exact match when the hypothesis is an example).

This methodology is general and valid for all the hypotheses including extreme situations:

In the case of an exact match between the query and an example, the hypothesis search results in a single hypothesis (the example itself) and the prediction reflects the factual data:

$$\mathrm{if}\;\exists e,e=x\;\mathrm{then}\;\mathrm{clas}s_{h,x}\;\mathrm{is}\;\mathrm{class}\left(e\right);\mathrm{confidenc}e_{h,x}\;\mathrm{is}\;1$$

Similarly, at the opposite extreme, there is always at least one hypothesis that applies to the query compound which is the root of the SOHN (which matches the whole chemical space). If no other hypothesis is found during the SOHN exploration the kNN is applied to the supporting examples of the root (h_0_) which corresponds to the whole reference dataset.

### Overall call (global prediction)

Once a prediction and a confidence level have been calculated for each of the relevant hypotheses we can construct an overall call based on these values. Different reasoning heuristics can be used depending on the use case. Our default reasoning algorithm simply weights each hypothesis according to its confidence level. Additionally two parameters ‘a’ and ‘b’ are introduced to control the signal threshold for equivocal prediction (a) and the balances between sensitivity and specificity (b). The overall signal s_x_ for the query compound can be calculated as the follows:

$$\begin{array}{c} s_{x}=\frac{\sum_{h=1}^{m}s_{h,x}\times \mathrm{confidenc}e_{\;h,x}}{\sum_{h=1}^{m}\mathrm{confidenc}e_{h,x}}\\ \mathrm{clas}s_{x}=\left\{\begin{array}{c} \mathrm{positive}\;\mathrm{if}\;s_{x}>a+b\\ \mathrm{negative}\;\mathrm{if}\;s_{x}<a-b\\ \mathrm{equivocal}\;\mathrm{if}\;a-b\leq s_{x}\leq a+b \end{array}\right)\\ \mathrm{confidenc}e_{x}=\left|s_{x}\right| \end{array}$$

**Figure 19:**
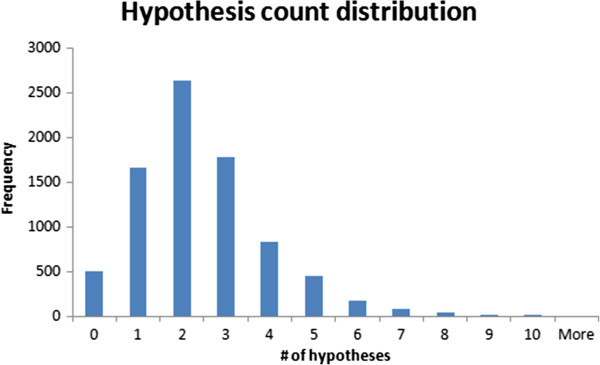
**Number of hypotheses used per prediction.** The figure presents a distribution of the hypothesis count per prediction during a 5 fold cross-validation experiment using the previous mutagenicity dataset (8201 structures). The most common case is a prediction based on 2 hypotheses and 90% of the predictions were based on 4 or less hypotheses.

Where:

• *m* is the number of relevant hypotheses

• *a* is the signal threshold for separating two classes (in a binary classification problem). The default value for *a* is 0

• *b* is a minimum level the signal must reach to become significant (otherwise it is considered as equivocal). The default value for *b* is 0.The number of hypotheses used for a given prediction should not be too important in order to maintain the interpretability of the model. Figure 19 shows the distribution of the number of hypotheses found per query during the 5-fold cross-validation experiment using the previously described mutagenicity dataset. From the figure we can conclude that the most common case is a prediction based on 2 hypotheses. A closer analysis reveals that 90% of the compounds were predicted using 4 or fewer hypotheses which favours an easy interpretation.

### Prediction confidence

**Figure 20:**
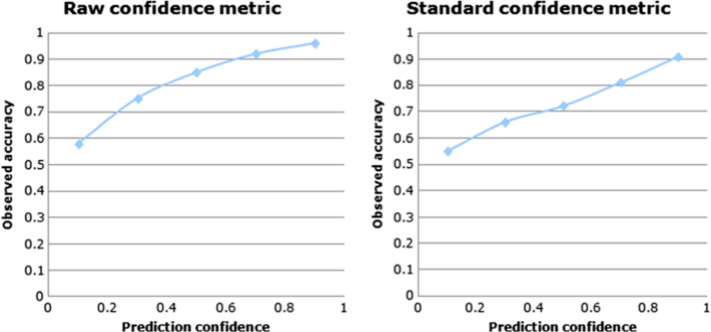
**Raw confidence vs. Standardised confidence.** Left, the raw confidence estimate and the actual observed accuracy correlate well but in a non linear way. Right, the standard confidence models has a more linear correlation with the observed accuracy after applying a polynomial fit and provides an intuitive and normalized estimate of the accuracy for each individual prediction.

The confidence metric was designed to assist the end user in a decision process and to facilitate the comparison of different models with the assumption that in each case there is a comparable correlation between confidence and accuracy. Ideally a confidence metric would have a linear correlation between the individual prediction accuracy estimate and the observed accuracy. The behaviour of this confidence measure has been assessed by plotting the prediction confidence value against the observed accuracy during a 5-fold cross-validation using the previously described dataset. The prediction results were merged and split into 5 equidistant confidence bins for which the corresponding accuracy was plotted (Figure 20, left). As expected the predictions with confidence towards 0 have accuracy close to a random predictor (close to 50%) and prediction with a high confidence towards 1 approach the performance of a perfect model (close to 100%).

Although the correlation between confidence and accuracy is positive and monotonic, it is not linear and in order to provide a more intuitive confidence measure (linearly correlated to the accuracy) the model’s confidence value is refitted using an order 3 polynomial transformation T. This transformation provides the final normalized confidence called *standard confidence* (Figure 20, right)

$$\mathrm{standard}\;\mathrm{confidenc}e_{x}=T\left(\mathrm{raw}\;\mathrm{confidenc}e_{x}\right)$$

The exact coefficients of this fit function T are dependent on the SOHN model and are evaluated using the confidence vs. accuracy data measured during cross validation.

The final overall prediction provides the following set of information:

• class_x_: predicted class

• *standard confidence*_x_: normalised confidence level for the prediction

• *{h}*: Set of hypotheses relevant to the query compound and which have been used in the overall call.

And for each hypothesis:

• Class_x,h_: The local class prediction for this hypothesis

• *confidence*_x_: the confidence associated with the hypothesis

• *{e}*: a set of examples supporting the hypothesis

As we can see the SOHN prediction methodology provides rich information for the end user to facilitate the assessment of the model’s conclusion. The expert can use the explanation inferred by the set of relevant hypothesis, integrate the confidence metric to establish a trust level and use the supporting examples (and their similarity with the query compound) to further refine the assessment. Finally using their own knowledge the experts can reject or confirm the model’s conclusion on the basis of a transparent prediction. Transparency was a major driver when developing this method.

### Applicability domain

When selecting relevant hypotheses the query compound has to be in the applicability domain of the retained hypotheses. Each type of hypotheses defines its own applicability domain (second clause of the contract); the domain should depend upon the type of information captured in this type of hypotheses. For instance, in our example of structural hypotheses, the applicability domain was defined based upon the dictionary of fragments constructed when fragmenting the reference dataset. In order to be inside the domain of applicability all parts of the query compounds must be covered by at least one ‘non rare’ fragment of the dictionary where ‘non rare’ means fragments occurring in at least 4 examples in the reference dataset.

The applicability domain is therefore delegated to the hypothesis types and this way we decouple the overall applicability domain management from the prediction algorithm. New types of hypotheses can be easily integrated in the methodology without having to revisit the prediction mechanism. If the applicability domain depends on a reference collection of structures it must be defined using the SOHN reference dataset since this is the chemical space used for the kNN prediction.

### Comparison with other methods

The SOHN methodology applied to the mutagenicity endpoint was compared to the Support Vector Machine (SVM), Random Forest (RF), k Nearest Neighbours (kNN) and Decision tree (DT) learning methods. For the hypotheses generation a recursive partitioning method (actually a simplified version of a decision tree as described in Figure 12) was used to identify fragments that yield the maximum information gain [27]. This experiment does not intend to directly compare quantitative performance since the actual knowledge extraction algorithm is a simplified decision tree for which we do not expect highly optimized performance compared to robust SVM and RF models. The goal is to verify that we can indeed decouple the learning method from the prediction algorithm using the SOHN approach without a significant loss of performance relative to the knowledge mining technique. Additionally we would like to observe the behaviour of the SOHN prediction algorithm in the case of a different chemical space compared to the other learning methods.

A dataset of public mutagenicity data was extracted from the Vitic database [28]. After curation the dataset contained 8,201 compounds divided into 4,152 mutagens (positives) and 4,049 non mutagens (negatives). In parallel, 5 times cross validations were performed using SOHN, SVN, RF, kNN and DT models for the same dataset and using the fragments generated in the fragmentation process as descriptors in order to use the same feature space.

**Figure 21:**
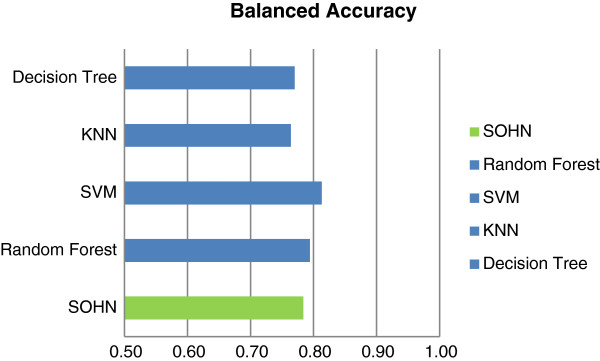
**Comparison of models for the public dataset.** The performance of the SOHN model (using recursive partitioning as the knowledge mining algorithm) is comparable to other mainstream machine learning methods.

Table 1 and Figure 21 show the results for the different models. The best model is obtained using the SVM algorithm followed by the Random Forest model. The SOHN model ranks third. Interestingly the Decision Tree and the kNN algorithms do not outperform the SOHN model which means that combining the Decision Tree approach for mining hypotheses and the SOHN/kNN method for predicting does not induce a loss of predictive performance. Although this is mainly a qualitative experiment, it is encouraging to see that the SOHN’s performance is comparable to other techniques and in addition provides transparent predictions.

**Table 1:** Comparison of models for the public dataset

**Cross validation using the public dataset (8201 structures)**					
**Fragments**	**BAC**	**SEN**	**SPEC**	**PPV**	**NPV**
SOHN	0.78	0.78	0.78	0.79	0.78
Random Forest	0.79	0.83	0.76	0.78	0.76
SVM	0.81	0.82	0.81	0.82	0.81
kNN	0.76	0.73	0.80	0.79	0.80
Decision Tree	0.77	0.76	0.79	0.79	0.79

BAC = balanced accuracy, SEN = sensitivity, SPEC = specificity, PPV = positive predictivity, NPV = negative predictivity.

A second experiment was run to analyse the behaviour of the SOHN model using a chemical space different from the training set. It is well known that toxicity prediction models trained on public data usually perform less well on proprietary data due to differences in chemical space coverage for reason of confidentiality [29]. Thanks to a data sharing initiative with several pharmaceutical companies we have had access to a dataset of intermediates used during the process of drug development. The chemical space represented by these compounds is specific to the pharmaceutical industry and it was interesting to analyse how different models project from the public training space onto this confidential test space. A model able to transpose from one chemical space to the other is especially valuable in the context of risk assessment.

**Figure 22:**
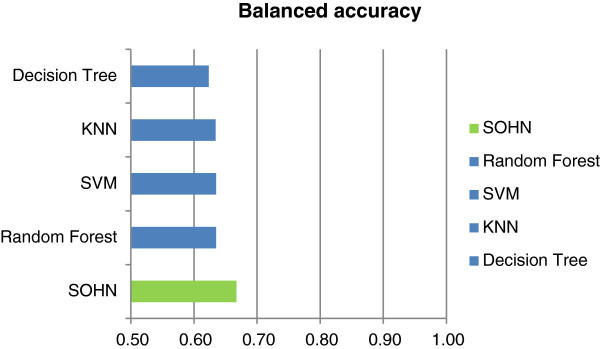
**Comparison of models for the confidential dataset.** The SOHN model’s performance is comparable to other methods and seems to be slightly more robust when applied to the confidential chemical space.

All 5 models were trained with the whole public dataset (8201) structures and the confidential intermediates dataset was used as a test set. The intermediates dataset contains 800 compounds of which only 30% are mutagens. It is a chemical space biased toward negative instances which adds difficulty to the prediction task. The same protocol as for the previous experiment was used. Table 2 and Figure 22 presents the results from this experiment. It can be seen that, as expected, all models perform less well on this challenging confidential chemical space. The SOHN model seems to be slightly more robust providing the best results in each category.

**Table 2:** Comparison of models for the confidential dataset

**External validation using the confidential dataset (800 structures)**					
	**BAC**	**SEN**	**SPEC**	**PPV**	**NPV**
SOHN	0.67	0.53	0.80	0.55	0.80
Random Forest	0.64	0.50	0.77	0.50	0.77
SVM	0.64	0.50	0.77	0.50	0.77
kNN	0.63	0.48	0.79	0.51	0.79
Decision Tree	0.62	0.45	0.80	0.50	0.80

BAC = balanced accuracy, SEN = sensitivity, SPEC = specificity, PPV = positive predictivity, NPV = negative predictivity.

**Figure 23:**
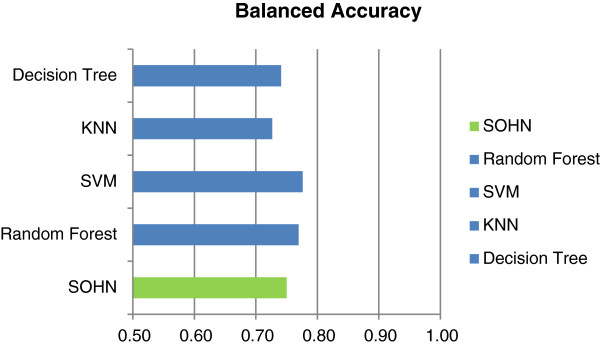
**Comparison of models for the CFSAN dataset.** The SOHN model’s performance is comparable to other methods on this dataset.

Finally, to complete this comparison, a second external validation was performed using a mutagenicity dataset provided by the Center for Food Safety and Applied Nutrition (CFSAN) [30]. Again, all 5 models were trained with the whole public dataset (8201) structures. The CFSAN dataset represents a chemical space used in a regulatory context and contains 1399 structure not duplicated in the training dataset. Like the intermediates dataset, the CFSAN chemical space is strongly biased toward negative instances (874 negative instances for 525 positive instances). Table 3 and Figure 23 indicate comparable performances between the different models.

**Table 3:** Comparison of models for the CFSAN dataset

**External validation using the FDA/CFSAN dataset (1399 structures)**					
	**BAC**	**SEN**	**SPEC**	**PPV**	**NPV**
SOHN	0.75	0.64	0.86	0.75	0.79
Random Forest	0.77	0.74	0.80	0.70	0.83
SVM	0.78	0.70	0.85	0.74	0.82
kNN	0.73	0.62	0.83	0.70	0.78
Decision Tree	0.74	0.62	0.87	0.74	0.78

Results for the confidential dataset. BAC = balanced accuracy, SEN = sensitivity, SPEC = specificity, PPV = positive predictivity, NPV = negative predictivity.

## Discussion

**Figure 24:**
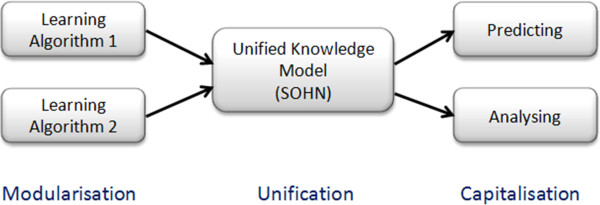
Overview of the SOHN approach.

The approach has successfully decoupled the learning phase from the prediction phase at the knowledge level by representing the knowledge in the form of hypotheses. While hypotheses were discovered using a recursive partitioning method, the corresponding Decision Tree was not used to perform the prediction. This separation did not lead to a loss of performance and actually even slightly improved it.The SOHN approach defines a consistent modular learning and prediction framework where learning methods can be interchanged or combined and where higher prediction or data analysis algorithms can be developed (Figure 24).

From the user’s viewpoint the SOHN approach results in a very transparent tool for knowledge discovery and prediction purposes. Early performance tests show that the approach provides results comparable to established methods even when using only 2 related sources of hypotheses (training examples and a simplified recursive partitioning to generate hypotheses from these examples). These results are very encouraging given the early stage of the development of this approach. The next step will be to combine different sources of knowledge (e.g. predefined set of structural static patterns like MACCS keys (Molecular ACCess System) or patterns mined by human experts) and merge the resulting knowledge into a single SOHN. From the prediction perspective we expect improved performance and from the SAR analysis viewpoint it will be interesting to visualise how the expert hypotheses are blended into the machine learned knowledge.

**Figure 25:**
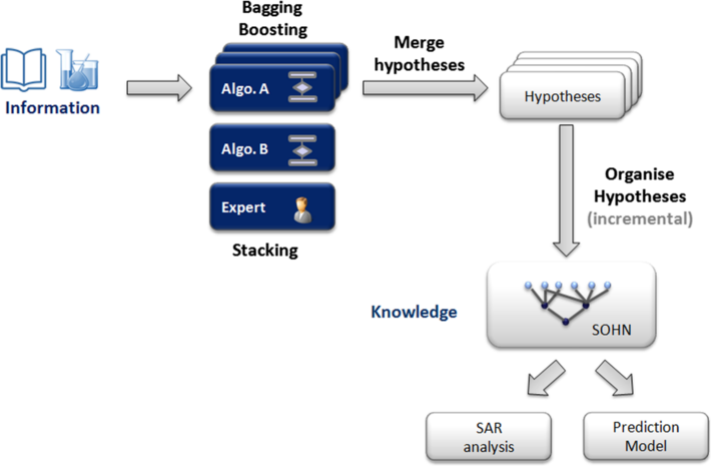
**Applying ensemble modelling to the SOHN.** Different learning methods and different iterations of bagging and boosting optimisation can contribute to the same SOHN in order to construct an extensive knowledge asset that can be used for accurate and transparent prediction or detailed SAR analysis.

Since the SOHN facilitates the combination of different learning methods it also provides a mechanism to build an ensemble of models at the knowledge level. Combining different knowledge sources into a single SOHN is a form of *stacking*[31], in this case “Knowledge stacking”. Similarly other ensemble techniques can be applied like *bagging*[32] and *boosting*[33] to further improve the knowledge extraction process. In this case each bagging or boosting iteration contributes independently to the SOHN. The resulting knowledge is automatically merged and organised into a single common SOHN for which we can apply the same prediction and SAR analysis tools, allowing the end user to access an optimised model for both predictive and knowledge discovery purposes (Figure 25).

Another key feature of the SOHN approach is to support different types of hypotheses. For the mutagenicity endpoint, only structural hypothesis were used; in future work, other important hypothesis types like physico-chemical or pharmacophoric hypotheses will be added to show how different kinds of knowledge units can be used by the prediction algorithm to build transparent and elegant conclusions. For instance we expect predictions in the form of:

“This compound is active because it contains this moiety (structural hypothesis) but the confidence is low owing to a high logP value (physico-chemical hypothesis). ”

**Figure 26:**
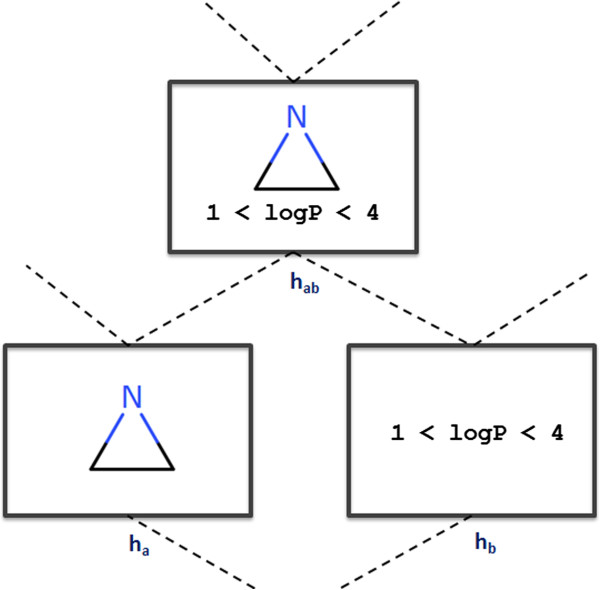
**Combining hypotheses.** The abstraction of the hypotheses permits new ways of combining knowledge units to be defined. Creating hypothesis combination operators is possible; in this figure hypothesis h_a_ and h_b_ are combined using a conjunction operator to produce h_ab_. If h_ab_ is a valuable unit of knowledge (e.g. provides enough information gain) then it can be kept and inserted in the knowledge. This is a form of meta-learning.

The SOHN framework allows this kind of inference even though both hypotheses may have been originated from different learning approaches.Finally, the formalisation and unification of the knowledge opens up the potential for combining the hypotheses within the SOHN to develop optimisation or repurposing algorithms independently. For instance, using the formal contract of hypotheses we can design operators to combine hypotheses regardless of their type (Figure 26). It is possible to develop algorithms to identify hypothesis associations that further improve the predictive performance of the SOHN and hence extend the learning process at the SOHN level (meta-learning). In this context SOHN can benefit from the techniques used in formal concept analysis. This powerful aspect of the SOHN will also be investigated in future work.

## Conclusion

We have presented a new approach for representing and organising knowledge in the form of Self Organising Hypothesis Networks. This method decouples the learning phase from the application phase by introducing an intermediate knowledge layer based on the concept of hypothesis. A hypothesis represents a simple and interpretable unit of knowledge which may describe any relevant aspect of a chemical compound in the context of an endpoint. Decoupling the learning and the application framework allows us to combine different sources of knowledge into the same formal framework and to apply mutualised algorithms. By introducing the concept of hypothesis we define a higher level model that is independent of the learning technique and operates at the early knowledge level rather than at the later prediction stage as is the case for consensus models. We have presented a simple application to the mutagenicity endpoint and shown that the SOHN model’s performances are comparable to other common type of models while showing improved robustness when testing with external validation sets. The predictions are transparent as the result of the interpretable nature of the hypotheses. The proposed SOHN prediction algorithm assigns a confidence level for each individual prediction; this accuracy estimate correlates well with that observed. The hierarchical structure of the SOHN also facilitates the identification of interesting SAR patterns and activity cliffs; it can be used as a powerful assistant in knowledge discovery and lead optimisation.

The SOHN methodology clearly offers further potential in the context of SAR analysis and for building accurate and transparent predictive modules. This article covers the general principle of this new paradigm and describes a formal framework to manage hypotheses and their application. Although initially focused on binary classification tasks, the underlying formal methodology should equally apply to multi class classification and regression problems. Upcoming research will explore the potential of combining different sources of knowledge, mixing different types of hypotheses and applying bagging and boosting optimisation techniques. Future work will also investigate the ability to extend the learning process inside the SOHN as a form of meta- learning.

## Abbreviations

CFSAN: Center for food safety and applied nutrition; DT: Decision tree; FCA: Formal concept analysis; ILP: Inductive logic programming; kNN: K nearest neighbours; MACCS: Molecular ACCess System; MMP: Matched molecular pair; OECD: Organisation for Economic Cooperation and Development; QSAR: Quantitative structure activity relationship; RF: Random forest; SAR: Structure activity relationship; SOHN: Self organizing hypothesis network; SVM: Support vector machine.

## Competing interests

We declare no direct competing interests; however the science presented in the article is used in a commercial product owned by the employer of the authors (i.e. Lhasa Limited).

## Authors’ contributions

TH: Designed the SOHN approach and contributed to its implementation. CB: Contributed to scientific elaboration of the SOHN approach. ER: Contributed to scientific elaboration and the implementation of the SOHN approach. JV: Contributed to scientific elaboration of the SOHN approach. SW: Contributed to scientific elaboration and the implementation of the SOHN approach, performed machine learning experiments. SJW: Contributed to scientific elaboration of the SOHN approach and performed machine learning experiments. All authors read and approved the final manuscript.
